# Monitoring Aircraft Position Using EGNOS Data for the SBAS APV Approach to the Landing Procedure

**DOI:** 10.3390/s20071945

**Published:** 2020-03-30

**Authors:** Kamil Krasuski, Damian Wierzbicki

**Affiliations:** 1 Institute of Navigation, Military University of Aviation, 08-521 Dęblin, Poland; k.krasuski@law.mil.pl; 2 Institute of Geospatial Engineering and Geodesy, Faculty of Civil Engineering and Geodesy, Military University of Technology, 00-908 Warsaw, Poland

**Keywords:** EGNOS, accuracy, continuity, integrity, availability, SBAS APV

## Abstract

The aim of this paper is to present the problem of the implementation of the EGNOS (European Geostationary Navigation Overlay Service) data for the processing of aircraft position determination. The main aim of the research is to develop a new computational strategy which might improve the performance of the EGNOS system in aviation, based on navigation solutions of an aircraft position, using several GNSS (Global Navigation Satellite System) onboard receivers. The results of an experimental test conducted by the Cessna 172 at EPDE (European Poland Deblin) (ICAO (International Civil Aviation Organization) code, N51°33.07’/E21°53.52’) aerodrome in Dęblin are presented and discussed in this paper. Two GNSS navigation receivers with the EGNOS positioning function for monitoring changes in the parameters of the aircraft position in real time during the landing phase were installed onboard a Cessna 172. Based on obtained research findings, it was discovered that the positioning accuracy was not higher than 2.1 m, and the integrity of positioning did not exceed 19 m. Moreover, the availability parameter was found to equal 1 (or 100%); also, no intervals in the continuity of the operation of the EGNOS system were recorded. In the paper, the results of the air test from Dęblin were compared with the parameters of positioning quality from the air test conducted in Chełm (ICAO code: EPCD, N51°04’57.8” E23°26’15”). In the air test in Chełm, the obtained parameters of EGNOS quality positioning were: better than 4.9 m for accuracy, less than 35.5 m for integrity, 100% for availability, and no breaks in continuity. Based on the results of the air tests in Dęblin and Chełm, it was concluded that the parameters of the EGNOS positioning quality in aviation for the SBAS (Satellite Based Augmentation System) APV (Approach to Vertical guidance) procedure were satisfied in accordance with the ICAO (International Civil Aviation Organization) requirements. The presented research method can be utilized in the SBAS APV landing procedure in Polish aviation. In this paper, the results of PDOP (Position Dilution of Precision) are presented and compared to the two air tests in Dęblin and Chełm. The maximum results of PDOP amounted to 1.4 in the air test in Dęblin, whereas they equaled 4.0 in the air test in Chełm. The paper also shows how the EGNOS system improved the aircraft position in relation to the only GPS solution. In this context, the EGNOS system improved the aircraft position from about 78% to 95% for each ellipsoidal coordinate axis.

## 1. Introduction

The SBAS (Satellite Based Augmentation System) augmentation systems have been used in aviation to improve aircraft positioning performance in real time. Among SBAS augmentation systems exploited in aviation, it is possible to distinguish the following: European EGNOS (European Geostationary Navigation Overlay Service), Russian SDCM (System for Differential Corrections and Monitoring), American WAAS (Wide Area Augmentation System), Japanese MSAS (Multi-functional Satellite Augmentation System), Indian GAGAN (GPS Aided Geo Augmented Navigation), and African ASAS (African Satellite Augmentation System). The use of SBAS systems in aviation is to improve the basic parameters of the GNSS (Global Navigation Satellite System) positioning quality in aviation, i.e., accuracy, integrity, continuity, and availability. In Polish aviation, the European EGNOS augmentation system will be used to execute air operations. Ultimately, the EGNOS satellite system will be used in airline operations such as SBAS APV-I (Approach with Vertical Guidance) and APV-II SBAS approaches to landing [1,2]. The performance and requirements for a particular type of approach, i.e., SBAS APV-I and APV SBAS-II, are shown in Table 1.

Air tests using the EGNOS system have been conducted within the research projects “BRDA” and “Odra” [4,5] since 2003 in Poland. Subsequent tests were carried out on the air test “LIWIEC” in 2007 [6]. Further air tests were conducted from 2010 to 2011 for aerodromes located in south-eastern Poland [7]. In 2013, experiments were conducted in eastern Poland [5]. In the research [4,5,6,7], the application of the EGNOS system was suggested to examine the accuracy of aircraft positioning in real time for the needs of air navigation. In air tests, the determined aircraft position was verified with a precise trajectory of the flight reference obtained from the RTK-OTF (Real Time Kinematic–On The Fly) differential technique. At the same time in Poland, work began on the use of the EGNOS system in the approach to landing system—SBAS APV. The air tests were conducted in 2011 at the Katowice, Kraków, and Mielec airports by the Polish Air Navigation Services Agency (PANSA) [8,9,10]. The works [8,9,10] proposed the development of an experimental approach to landing procedures based on EGNOS and GNSS. Therefore, aircraft position on the approach to the landing path was examined in order to conduct navigation in the LNAV (Lateral Navigation) horizontal and VNAV (Vertical Navigation) vertical planes. Another direction of research with the use of the EGNOS system in Polish aviation was to determine the integrity of satellite positioning. In the works [11,12,13], the use of the EGNOS system was suggested to define the safety levels of the integrity of HPL (Horizontal Protection Level) and VPL (Vertical Protection Level) positioning during air test and during continuous monitoring at aerodromes. The experimental tests were carried out at the aerodromes in Dęblin and Olsztyn Dajtki. Moreover, a very interesting solution of using the EGNOS system in the Polish Air Force is used to estimate the average errors of determining a user position. These research tests were conducted for the area at selected airports in Warsaw, Rzeszów, Gdynia, and Kraków [14].

Throughout the world, the EGNOS system has been applied in the fields of navigation, transport, and aeronautical engineering. In [15], the use of EGNOS positioning was proposed for examining the accuracy of aircraft navigation in the framework of navigation procedures for the SBAS APV-I procedure. In turn, in [16], the use of the EGNOS system was proposed for examining the availability parameter under the ESTB program (EGNOS System Test Bed). However, in [17], an examination of the EGNOS performances for the LPV (Localizer Performance with Vertical Guidance) 200 minimum approach and of the system operation within the RNAV (Area Navigation) GNSS area navigation was proposed. Another solution proposed in [18] was to investigate the performance of the EGNOS system within the PA (Precision Approach) cat.I approach to the landing procedure, and later to compare it with the ILS (Instrumental Landing System) system findings. In the works [19,20,21], the authors proposed the investigation of HPE (Horizontal Position Error) and VPE (Vertical Position Error) positioning errors and the HPL (Horizontal Protection Level) and VPL (Vertical Protection Level) integrity levels in air operations in air transport.

It should be noted that monitoring the operation of the EGNOS system was conducted in air tests with at least one or two GNSS receivers in Poland and abroad. However, the position and the remaining navigation parameters of the aircraft were determined separately for each of the GNSS receivers mounted on board. Therefore, there was no combination or joining of EGNOS positioning solutions from two or more GNSS receivers in air operations. Such a combination of navigation solutions is innovative from the perspective of the implementation of the EGNOS system in Polish aviation. A unique approach to solving a research problem for Polish aviation by monitoring aircraft position by means of the EGNOS system based on the operation of two onboard GNSS receivers mounted on the aircraft is presented in this paper. In addition, the paper proposes experimental scientific tests relating to the designation of an aircraft position in the SBAS APV approach to the landing procedure, which emphasizes the quality of the obtained findings. The novelty is focused on a combination of individual solutions of aircraft positions for designating the resulting value of coordinates based on EGNOS data. In addition, the new method of research is presented in the paper. Finally, the parameters of accuracy, integrity, continuity, and availability were estimated for the resultant position of the aircraft based on the EGNOS solution. The main objective of the paper was achieved through research in two air tests in Dęblin and Chełm.

## 2. Research Method and Experimental Data

Mounting two or more GNSS receivers on board the aircraft can create a combination of solutions within the operation of the EGNOS system in aviation. Therefore, it is possible to designate the resulting position of aircraft based on individual solutions for the EGNOS system from several independent GNSS receivers. Within the EGNOS augmentation system, aircraft position is determined based on the mathematical model of the SPP (Single Point Positioning) positioning method for GPS code observations [22]. In addition, in the mathematical model of the observation equation in the SPP methods, long-term and fast EGNOS corrections are taken into consideration. In this way, it is possible to correct the basic parameters of the observation model in the GPS navigation system, i.e., the terms of a satellite position in an orbit, the term of satellite clock corrections, the ionospheric correction term, and the tropospheric correction term [23]. By using the above-mentioned parameter models, the EGNOS system facilitates an improvement of navigation performances of the GPS system in aviation. The basic equation of the SPP method for EGNOS corrections in GPS system can be presented as follows [24]:

(1) l=d∗+c⋅(dtr−dts∗)+Ion∗+Trop∗+Rel+TGD+PRC+Mp,

where:

l—pseudo-range in GPS system,
$d^{*}$—geometric distance satellite-receiver, the GPS satellites coordinates are modified by long-term EGNOS corrections,
c—speed of light,
dtr—receiver clock bias,
$dts^{*}$—satellite clock bias, parameter is modified by long-term EGNOS corrections,
$Ion^{*}$—ionosphere correction, parameter is estimated by using the SBAS grid model,
$Trop^{*}$—troposphere correction, parameter is estimated by using RTCA–MOPS (Radio Technical Commission for Aeronautics-Minimum Operational Performance Standards) model,
Rel—relativistic effect,
TGD—timing group delay,
PRC—EGNOS fast corrections,
Mp—multipath effect.

From Equation (1), the aircraft position in (φ—latitude, λ—longitude, h—ellipsoidal height) ellipsoidal coordinates for each GNSS receiver are calculated for each measurement epoch. Thus, the resultant position of an aircraft in the EGNOS solution, from several individual GNSS receivers, can be expressed as follows [4]:

(2) $$\{\begin{array}{l} \varphi_{m}=\frac{\varphi_{1}+\ldots +\varphi_{N}}{N}\\ \lambda_{m}=\frac{\lambda_{1}+\ldots +\lambda_{N}}{N}\\ h_{m}=\frac{h_{1}+\ldots +h_{N}}{N} \end{array},$$

where:

$\varphi_{m}$—an average value of an aircraft position along the latitude axis from the EGNOS solution,
$\lambda_{m}$—an average value of an aircraft position along the longitude axis from the EGNOS solution,
$h_{m}$—an average value of an aircraft position along the ellipsoidal height axis from the EGNOS solution,
$\varphi_{1}$—an estimated aircraft position from receiver number 1 along the latitude axis from the EGNOS solution,
$\varphi_{N}$—an estimated aircraft position from receiver N along the Latitude axis from the EGNOS solution,
$\lambda_{1}$—an estimated aircraft position from receiver number 1 along the longitude axis from the EGNOS solution,
$\lambda_{N}$—an estimated aircraft position from receiver N along the longitude axis from the EGNOS solution,
  $h_{1}$—an estimated aircraft position from receiver number 1 along the ellipsoidal height axis from the EGNOS solution,
$h_{N}$—an estimated aircraft position from receiver N along the ellipsoidal height axis from the EGNOS solution,
N—a number of onboard GNSS receivers.

For the resultant position of an aircraft, there are also standard deviations of the ellipsoidal coordinates, as below [25,26]:

(3) $$\{\begin{array}{l} \delta \varphi_{m}=\sqrt{\frac{\left[d\varphi^{2}\right]}{N-1}}\\ \delta \lambda_{m}=\sqrt{\frac{\left[d\lambda^{2}\right]}{N-1}}\\ \delta h_{m}=\sqrt{\frac{\left[dh^{2}\right]}{N-1}} \end{array}$$

where:

$\delta \varphi_{m}$—a standard deviation of an average aircraft position along the latitude axis from the EGNOS solution,
$\delta \lambda_{m}$—a standard deviation of an average aircraft position along the longitude axis from the EGNOS solution,
$\delta h_{m}$—a standard deviation of an average aircraft position along the ellipsoidal height axis from the EGNOS solution,
$d\varphi =\varphi_{i}-\varphi_{m}$—a position error along the latitude axis,
$\varphi_{i}=\left[\varphi_{1},\ldots ,\varphi_{m}\right]$—an aircraft position along the latitude axis for each individual GNSS receiver,
$d\lambda =\lambda_{i}-\lambda_{m}$—a position error along the longitude axis,
$\lambda_{i}=\left[\lambda_{1},\ldots ,\lambda_{m}\right]$—an aircraft position along the longitude axis for each individual GNSS receiver,
$dh=h_{i}-h_{m}$—a position error along the ellipsoidal height axis,
$h_{i}=\left[h_{1},\ldots ,h_{m}\right]$—an aircraft position along the ellipsoidal height axis for each individual GNSS receiver.

Equation (3) is based on the fact that models of the ionosphere, troposphere, orbits, and clocks are the same in each single SBAS solution. The relation between a single solution of SBAS in relation to average position is presented in Chapter 4 “Discussion” (see Figures 12–14).

Based on the determined standard deviations of ellipsoidal coordinates, the integrity of EGNOS positioning was designated in aviation in the form of HPL and VPL parameters, as below [27]:

(4) $$\{\begin{array}{c} HPL=K_{H}\cdot \sqrt{\delta \varphi_{m}^{2}+\delta \lambda_{m}^{2}}\\ VPL=K_{V}\cdot \delta h_{m} \end{array},$$

where:

HPL—a horizontal protection level of an average aircraft position from the EGNOS solution,
VPL—a vertical protection level of an average aircraft position from the EGNOS solution,
$K_{H}$—a coefficient for SBAS APV procedure for landing in a horizontal plane, $K_{H}=6.00$,
$K_{V}$—a coefficient for SBAS APV procedure for landing in a vertical plane, $K_{V}=5.33$.

The investigation also specified the values of availability parameters of the navigation solution in the EGNOS system, in the approach to the landing procedure, as below [28]:

(5) $$A=1-\frac{t_{accident}}{t_{all}},$$

where:

A—an availability term of average aircraft position from the EGNOS solution,
$t_{accident}$—time of accident, time without solution from the EGNOS system,
$t_{all}$—time of all observations from the EGNOS solution.

In addition, the continuity parameter of the navigation solution of a position was made, in the EGNOS system, in the approach to landing procedure, as below [28]:

(6) $$C=1-\frac{t_{operation}}{t_{break}+t_{operation}},$$

where:

C—a continuity term of an average aircraft position from the EGNOS solution,
$t_{operation}$—time of system operation from the EGNOS solution,
$t_{break}$—time of system missing the solution from the EGNOS.

In the case of the continuity parameter of a navigation solution of a position in the EGNOS system, the number of intervals in which aircraft coordinates are not determined is not specified. The values of the continuity parameter are determined both in the SBAS APV-I and SBAS APV-II procedure in the EGNOS solution.

The mathematical Equations (1)–(6) were tested in the framework of the aviation experiment executed by the Cessna 172 at EPDE (European Poland Deblin) aerodrome in Dęblin. Within the air test, it was possible to determine the position of the Cessna 172 in the EGNOS solution. Two GNSS satellite receivers with the function of registration and tracking down the EGNOS system in real time were mounted on board the aircraft. Single frequency receiver Thales Mobile Mapper (Thales company, Massy, France), and a dual-frequency receiver Topcon HiperPro (Topcon company, Tokyo, Japan) were fixed in the cockpit. The antennas of GNSS receivers were mounted in the cockpit at a distance of less than 10 cm from each other [25]. Based on this, it was possible to designate the Cessna 172 position, from the EGNOS solution, for each of the mentioned GNSS receivers. The mathematical method of determining the Cessna 172 position was the method of SPP code positioning with a function of using EGNOS differential corrections (see Equation (1)). The obtained solutions of the Cessna 172 position from two independent receivers allowed the determination of the resultant values of the coordinates during the flight. The resultant position of the Cessna 172 in the EGNOS solution was determined in the ellipsoidal coordinates in accordance with the mathematical Equation (2). In turn, the standard deviations of the resultant aircraft position were determined in accordance with Equation (3). Moreover, the parameters of integrity, availability, and continuity of solving the aircraft position in the EGNOS system were determined based on mathematical Equations (4), (5), and (6), respectively.

**Figure 1:**
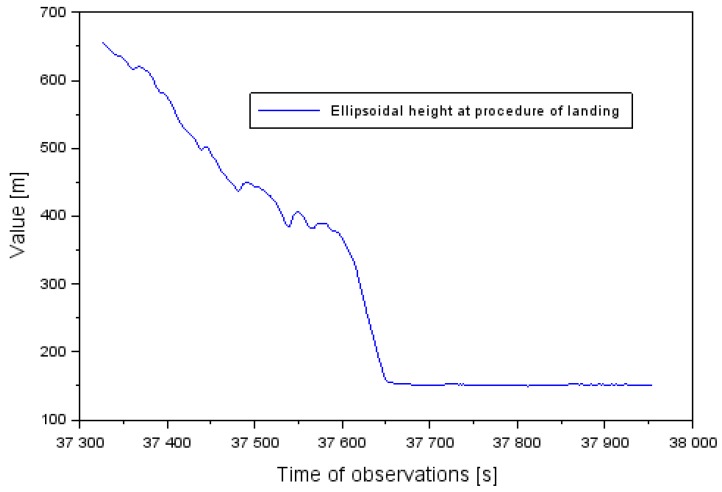
The vertical profile of Cessna 172 aircraft at landing procedure at the Dęblin aerodrome.

Examination of the position of the Cessna 172 and the quality of EGNOS positioning was conducted in the approach to the landing phase, within the SBAS APV procedure. During this time, the Cessna 172 approached to land at the EPDE military aerodrome in Dęblin, in the south direction on runway RWY30 (Run Way 30) [29]. In addition, the altitude of the Cessna 172 varied from 655 to 152 m (see Figure 1). The descent time of the Cessna 172 before landing at the Dęblin aerodrome equaled approximately 10.5 min. At the same time, the latitude coordinates of the aircraft ranged between 51.403307° to 51.556445° and for longitude between 21.868859° to 21.934283°.

## 3. Research Results

**Figure 2:**
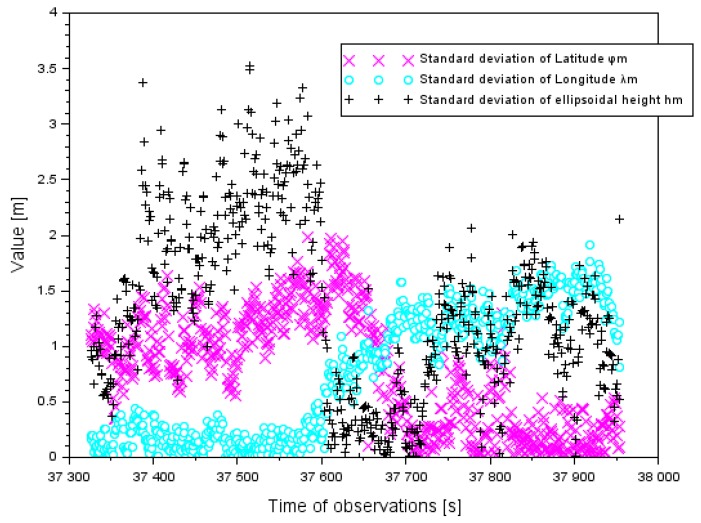
Standard deviation of an average aircraft position in the air test in Dęblin.

Within the experimental tests, a series of computer calculations for the proposed positioning EGNOS method in aviation were made. In the first step, standard deviations of the resultant Cessna 172 positions were determined, in accordance with Equation (2). The values of a standard deviation for the components $\left(\varphi_{m},\;\lambda_{m},h_{m}\right)$ were determined for the approach to landing phase of the Cessna 172. The standard deviation for the component $\varphi_{m}$ ranged between 0.1 and 2.0 m. Furthermore, the average value of the parameter $\delta \varphi_{m}$ was 0.8 m and the median was 0.8 m. The value of the standard deviation for the component $\lambda_{m}$ ranged between 0.1 and 1.9 m. The average value of the parameter $\delta \lambda_{m}$ was 0.8 m and the median was also 0.8 m. The value of the standard deviation for the component $h_{m}$ was between 0.1 and 3.6 m. Moreover, the average value of the parameter $\delta h_{m}$ was 1.4 m and the median was 1.3 m. The obtained values of the standard deviation for the components $\left(\varphi_{m},\lambda_{m},h_{m}\right)$ are presented in Figure 2.

**Figure 3:**
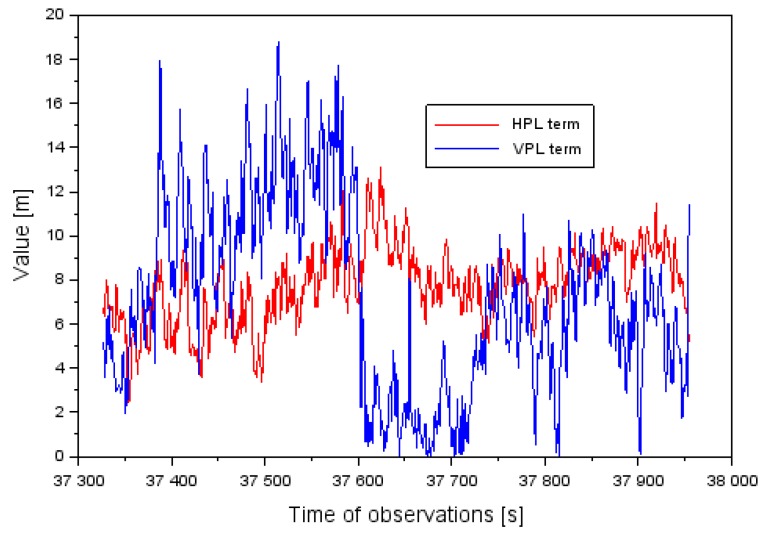
The HPL (Horizontal Protection Level) and VPL (Vertical Protection Level) values in the air test in Dęblin.

Figure 3 shows the obtained values of the levels of HPL and VPL integrity for the approach to landing aircraft phase of the Cessna 172. The values of the HPL and VPL parameters were determined based on Equation (3). The value of the HPL level ranges from 2.4 to 13.1 m. Furthermore, the average value of the HPL parameter equaled 7.8 m, and the median was 7.9 m. The value of the VPL level ranged between 0.1 and 18.8 m. Moreover, the average value of the VPL parameter equaled 7.2 m, and the median equaled 6.8 m. It should be noted that approximately 93% of all the HPL performance parameters were less than 10 m. On the other hand, over 96% of all the results of the VPL parameter were under 15 m.

Table 2 presents the results of the availability and continuity parameters in the EGNOS solution during the approach to the landing phase within the SBAS APV procedure. The values of the availability and continuity parameters in the EGNOS solution were determined from Equations (4) and (5). The value of the availability parameter in the SBAS APV-I and APV-II procedures during the approach to landing equaled A=1 (or 100%). The theoretical number of the EGNOS system failures equaled C=0.000042÷0.000335. However, for the analyzed time interval, there were no EGNOS system failures; therefore the availability and continuity of the SBAS system operation was preserved during the approach to landing by the Cessna 172.

**Figure 4:**
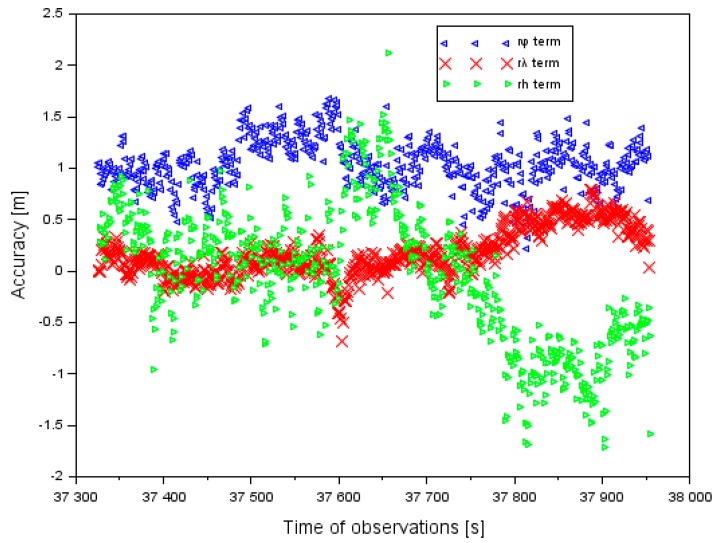
Accuracy of aircraft positioning in the ellipsoidal coordinates in the air test in Dęblin.

Figure 4 shows the EGNOS positioning accuracy during the approach to landing by the Cessna 172. The positioning accuracy values of the Cessna 172 in the ellipsoidal coordinates were defined as below [30]:

(7) $$\{\begin{array}{l} r\varphi =\varphi_{m}-\varphi_{PPP}\\ r\lambda =\lambda_{m}-\lambda_{PPP}\\ rh=h_{m}-h_{PPP} \end{array},$$

where:

$\left(\varphi_{m},\lambda_{m},h_{m}\right)$—an average position of the aircraft in the ellipsoidal coordinates from the EGNOS solution (see Equation (1)),
$\varphi_{PPP}$—the reference value of latitude from the PPP (Precise Point Positioning) solution,
$\lambda_{PPP}$—the reference value of longitude from the PPP solution,
$h_{PPP}$—the reference value of ellipsoidal height from the PPP solution.

The reference coordinates of the Cessna 172 position during landing were determined from the measurement technique PPP (Precise Point Positioning) for dual-frequency GPS observations. It is necessary to note that the mean errors of the Cessna 172 position, in the PPP solution, were approximately 10 cm in the approach to the landing phase [31]. The calculations of the reference position of the Cessna 172 were made in the GAPS (GPS Analysis and Positioning Software) program, developed at UNB (University of New Brunswick) in Canada [32]. The basic equation of the PPP method is described as follow [24]:

(8) {P3=d+c(dtr−dts)+Trop+Rel+MP3L3=d+c(dtr−dts)+Trop+B3+Rel+δw+ML3{P3=αP1+βP2=d+c(dtr−dts)+mfH⋅ZHD+mfW⋅ZWD+Rel+MP3L3=αL1+βL2=d+c(dtr−dts)+mfH⋅ZHD+mfW⋅ZWD+B3+Rel+δw+ML3,

where:

*P*_3_—Iono-free linear combination for GPS code observations,
*L*_3_—Iono-free linear combination for GPS phase observations,
*P*_3_ = α·*P*_1_ + β·*P*_2_,
*L*_3_ = α·L_1_ + β·L_2_,
*P*_1_—GPS code measurement at frequency *L*1,
*P_2_*—GPS code measurement at frequency *L*2,
*L*_1_—GPS phase measurement at frequency *L*1,
*L*_2_—GPS phase measurements at frequency *L*2,
*α* = +*f*_1_^2^/(*f*_1_^2^ – *f*_2_^2^),
*β* = –*f*_2_^2^/(*f*_1_^2^ – *f*_2_^2^),
(*f*_1_, *f*_2_)—GPS frequencies,
*d*—geometric distance satellite-receiver in the PPP method,
*c*—speed of light,
*dtr*—receiver clock bias,
*dts*—satellite clock bias,
*Trop*—troposphere delay,
*Trop* = *SWD* + *SHD*,
*SHD*—Slant hydrostatic delay,
*SWD*—Slant wet delay,
*SHD* = *mf_H_*·ZHD,
*SWD* = *mf_W_*·ZWD,
(*mf_H_*, *mf_W_*)—mapping function for hydrostatic and wet component,
*ZHD*—Zenith hydrostatic delay,
*ZWD*—Zenith wet delay,
Re*l*—relativistic effect,
*B*_3_—float phase ambiguity,
*δ_w_*—phase wind up,
*M_P_*_3_—multipath for code measurements,
*M_L_*_3_—multipath for phase measurements.

The results of the positioning accuracy of the Cessna 172, in the EGNOS solutions in the ellipsoidal frame, are shown in Figure 4. The positioning accuracy of the Cessna 172 for the component φ ranged between +0.2 and +1.7 m. In addition, the mean value of parameter rφ equaled +1.0 m, with an RMS error of approximately 1.1 m. The mean value of the positioning accuracy of the Cessna 172 for the component λ equaled +0.2 m and for the RMS error it was 0.3 m. Furthermore, the order of magnitude of the results obtained for the difference in the coordinates along the axis λ ranged from −0.7 to +0.8 m. The mean value of the positioning accuracy of the Cessna 172 for the component *h* equaled −0.1 m, whereas the RMS (Root Mean Square) error equaled 0.7 m. In addition, the amplitude of the obtained results of the positioning accuracy along axis *h* ranged between −1.7 and +2.1 m. The lowest positioning accuracy of the Cessna 172 was noticeable along axis *h*, and the highest accuracy was for the coordinates φ and λ. Furthermore, the RMS error was the largest for coordinate φ and the lowest for coordinate λ.

## 4. Discussion

**Figure 5:**
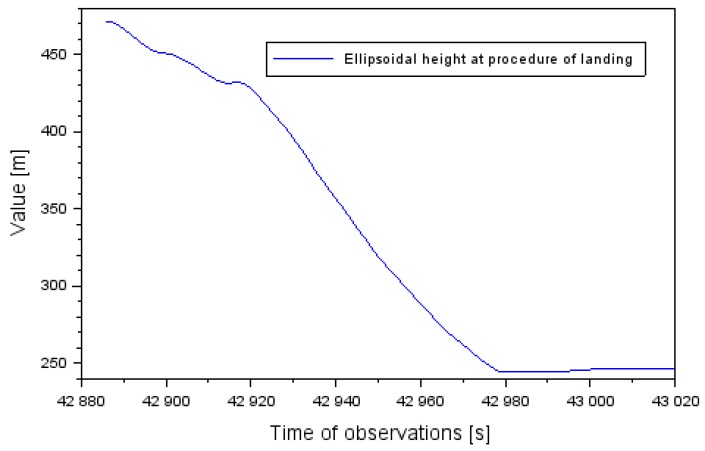
Vertical profile of the Cessna 172 during the landing process at Chełm airport.

The results of monitoring the changes in parameters of EGNOS positioning quality for the air test in Dęblin were compared with the navigation performances for the airborne air test conducted in Chełm, in south-eastern Poland. The air test in Chełm was also conducted by the Cessna 172 at the Depułtycze Królewskie aerodrome (ICAO (International Civil Aviation Organization) code: EPCD) [33]. Two satellite GNSS receivers with the EGNOS positioning function were mounted on board the Cessna 172. Thales Mobile Mapper (Thales company, Massy, France) and Javad Alpha (Javad company, San Jose, CA, USA) receivers were installed. Based on mathematical algorithms from Equation (1) to (6), the quality of monitoring changes in the Cessna 172 position during an approach to landing at the aerodrome in Chełm was determined. The altitude of the flight of the Cessna 172 during an approach to landing at Chełm aerodrome changed from 462 to 247 m (see Figure 5). The landing time of the Cessna 172 in Chełm exceeded 2 min. At the same time, the latitude coordinates of Cessna 172 aircraft ranged between 51.082398° to 51.102435°^o^ and for longitude between 23.436766° to 23.469734°, respectively.

**Figure 6:**
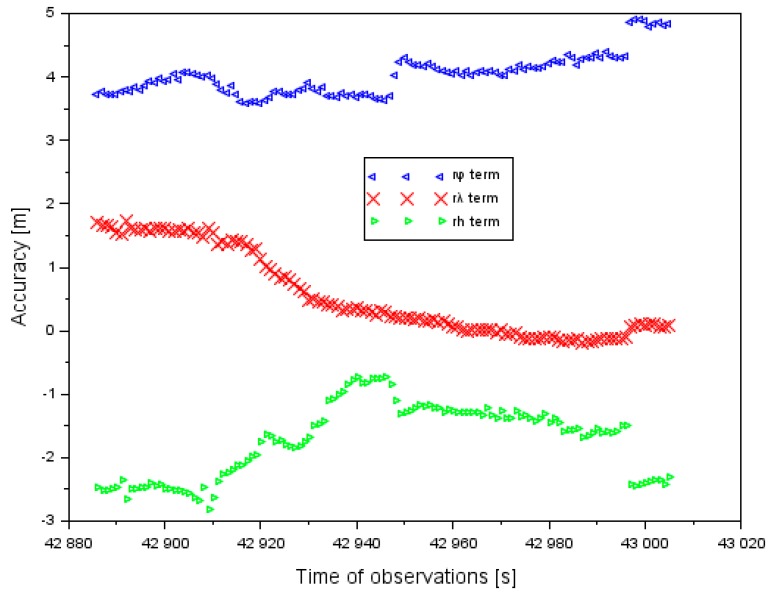
The accuracy of aircraft positioning in ellipsoidal coordinates during the air test in Chełm.

Figure 6 shows the results of the EGNOS positioning accuracy in the air test in Chełm. The values of the positioning accuracy of the Cessna 172 were determined based on Equation (6). The real position of the Cessna 172 in the airborne air test was determined based on the PPP measurement technique in the GAPS program.

The positioning accuracy of the Cessna 172 along the component φ ranged between +3.6 and +4.9 m. In addition, the mean value of parameter rφ equaled +4.0 m, with an RMS error of approximately 4.0 m. The mean value of the positioning accuracy of the Cessna 172 along the component λ equaled +0.6 m, and the RMS error was 0.9 m. Furthermore, the order of magnitude of the results obtained for the difference in the coordinate along the axis λ ranged between −0.2 and +1.7 m. The mean value of the positioning accuracy of the Cessna 172 for the component *h* equaled −1.7 m, whereas the RMS error equaled 1.8 m. The amplitude of the obtained results of positioning accuracy along the axis h ranged between −2.8 and −0.7 m. The lowest positioning accuracy of the Cessna 172 was noticeable along the axis φ, and the highest accuracy was for the coordinate λ.

Table 3 shows a comparison of the positioning accuracy of the Cessna 172 in the air test in Dęblin and Chełm. It needs to be emphasized that the accuracy of the positioning of the Cessna 172 in the horizontal LNAV plane was higher in the air test in Dęblin than in the air in Chełm. In addition, the positioning accuracy of the Cessna 172 in the VNAV plane was higher in the Dęblin air test than in the Chełm air test. Therefore, the obtained EGNOS positioning accuracy at the aerodrome in the air test in Dęblin was higher than in the Chełm air test.

**Figure 7:**
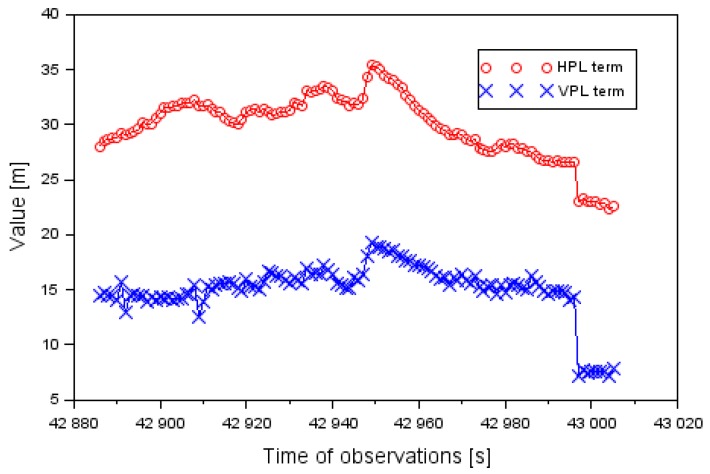
The HPL and VPL values of the air test in Chełm.

Figure 7 shows the obtained values of the levels of HPL and VPL integrity levels for the approach to the landing phase by the Cessna 172. The values of the HPL and VPL parameters were determined based on Equation (3). The value of the HPL level ranged between 22.3 and 35.5 m. Furthermore, the average value of the HPL parameter was 29.9 m. The value of the VPL level ranged from 7.1 to 19.2 m. The average VPL value equaled 15.1 m. The average difference between HPL and VPL values was around 15 m. In practice, the results of the HPL term were two times higher than VPL values. In addition, the obtained results of standard deviation of horizontal coordinates had a major impact on the estimation of the HPL parameter.

Table 4 shows a comparison of integrity positioning of the Cessna 172 in the air tests in Dęblin and Chełm for HPL and VPL parameters. It should be emphasized that the positioning integrity of the Cessna 172 in the horizontal LNAV plane was higher in the air test in Dęblin than in the air test in Chełm. In addition, the positioning integrity of the Cessna 172 in the VNAV plane was higher in the Dęblin air test than in the air test conducted in Chełm. Therefore, the positioning EGNOS integrity of the air test in Dęblin was higher than in the Chełm air test.

**Figure 8:**
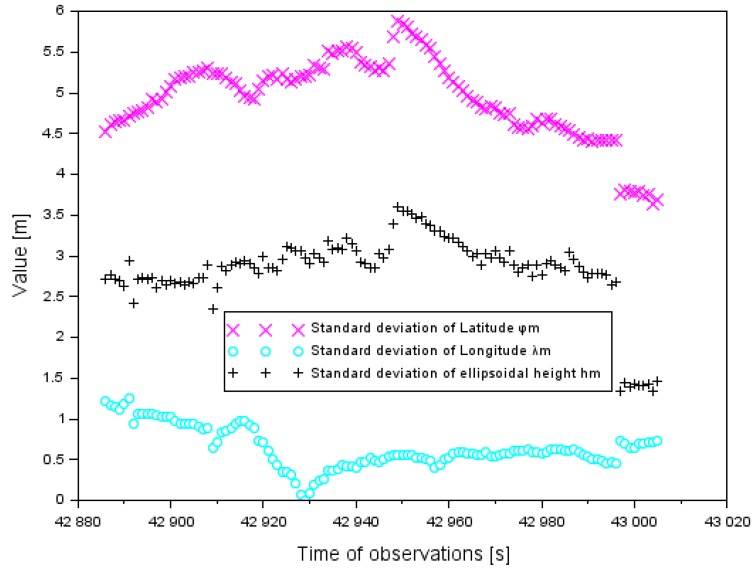
Standard deviation of an average aircraft position in the air test in Chełm.

In Figure 8, there are the results of the standard deviation analysis for the components $\left(\varphi_{m},\lambda_{m},h_{m}\right)$ of the Cessna 172 position for the air test in Chełm. The standard deviation for the components $\left(\varphi_{m},\lambda_{m},h_{m}\right)$ was designated in accordance with Equation (2), for the approach to landing phase by the Cessna 172. The value of standard deviation for component $\varphi_{m}$ ranged between 3.6 and 5.9 m. Furthermore, the average value of the parameter $\delta \varphi_{m}$ equaled 4.9 m. The value of the standard deviation for the component $\lambda_{m}$ ranged between 0.1 and 1.2 m. The average value of the parameter $\delta \lambda_{m}$ was 0.6 m. The standard deviation for the component $h_{m}$ ranged between 1.3 and 3.6 m. The average value of the parameter $\delta h_{m}$ was 2.8 m.

Table 5 shows a comparison of the standard deviations of the Cessna 172 coordinates in the air tests in Dęblin and Chełm. It should be emphasized that the parameter values $\delta \varphi_{m}$ were lower in the experimental test in Dęblin than in Chełm. On the other hand, the parameter values $\delta \lambda_{m}$ were smaller in the research test in Chełm than in Dęblin. The dispersion of results of parameter $\delta h_{m}$ was larger in the air test in Dęblin than for the data in the Chełm experiment.

Table 6 presents the results of the availability and continuity parameters in the EGNOS solution during the approach to the landing phase within the SBAS APV procedure at the aerodrome in Chełm. The values of the availability and continuity parameters in the EGNOS solution were determined in Equations (4) and (5). The value of the availability parameter in the SBAS APV-I and APV-II procedures during the approach to landing was equal to A=1 (or 100%). The theoretical number of the EGNOS system failures equaled C=0.000008÷0.000064. However, for the analyzed time interval, there was no EGNOS system failure, therefore the availability and continuity of the SBAS system operation was preserved during the approach to landing by the Cessna 172.

In the next part of the discussion, the results of PDOP (Position Dilution of Precision) geometric coefficients were analyzed for the air tests in Dęblin and Chełm (see Table 7). In the air test in Dęblin, the range of PDOP was between 0.9 and 1.4, with a mean value of 1.1. However, in the air test in Chełm, the values of PDOP ere higher, e.g., from 2.8 to 4.0. The values of PDOP in the air test in Chelm could have impacted the accuracy and integrity results. Particularly, it can be one of the reasons that the accuracy and integrity results were worse than in the air test in Dęblin. In GNSS satellite measurements, the value of the PDOP coefficient for which measurements were still being carried out in the field was 6. The obtained PDOP results were smaller than the value of 6, so the presented research method can be used in practice.

**Figure 9:**
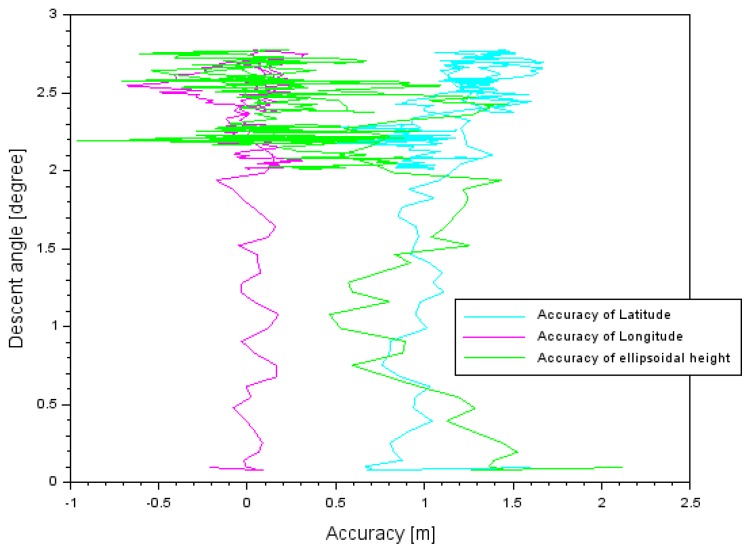
The descent angle in relation to accuracy results in the air test in Dęblin.

**Figure 10:**
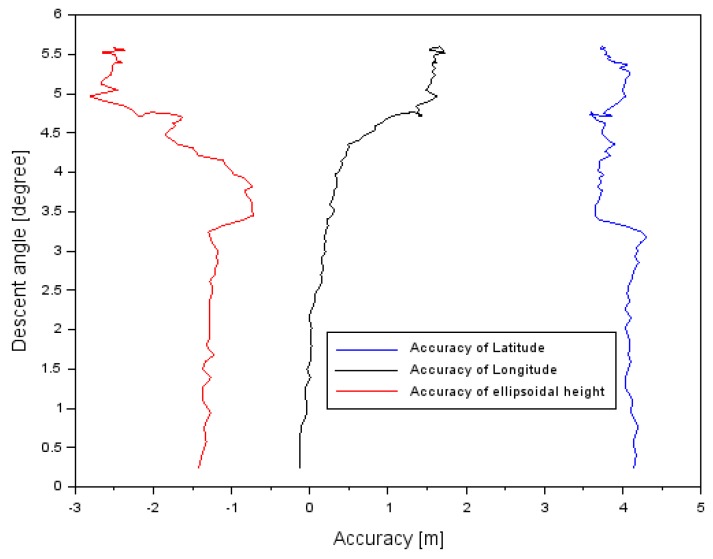
The descent angle in relation to accuracy results in the air test in Chełm.

The obtained accuracy was also compared with the descent angle during approach to landing procedure. The relationship between descent angle and accuracy in the air tests in Dęblin and Chełm is presented in Table 8 and in Figure 9 and Figure 10. In the air test in Dęblin, the descent angle changed between 0.1° and 2.7°. If the descent angle was around 0.1° before landing, then accuracy equaled 0.5 m for latitude, −0.7 m for longitude, and −1.0 m for ellipsoidal height. If the descent angle was about 2.7° at the initial path to landing, then accuracy equaled 1.7 m for latitude, +0.4 m for longitude, and +2.1 m for ellipsoidal height. In the air test in Chełm, the descent angle changed between 0.2° and 5.6°. If the descent angle was about 0.2° before landing, then accuracy equaled 3.6 m for latitude, −0.2 m for longitude, and −2.8 m for ellipsoidal height. If the descent angle was about 5.6° at the initial path to landing, then accuracy equaled 4.3 m for latitude, +1.7 m for longitude, and −0.7 m for ellipsoidal height. The path of landing was different for the air tests in Dęblin and Chełm and it also impacted accuracy results in both experiments.

**Figure 11:**
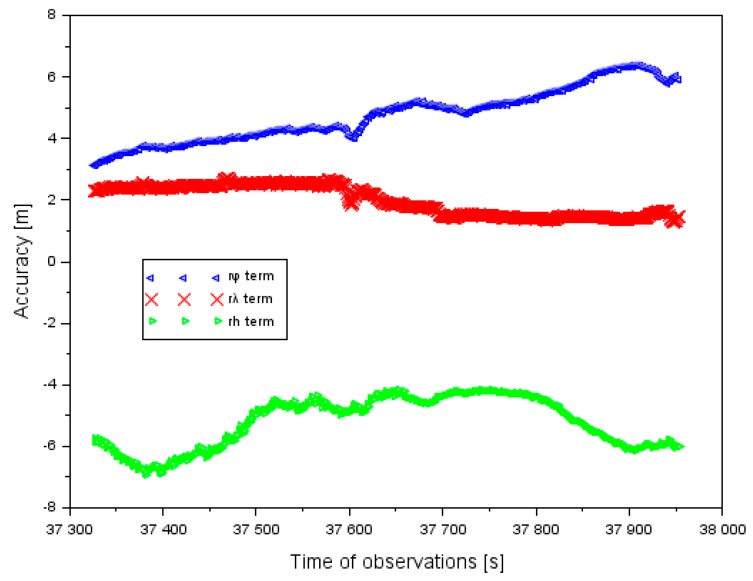
The accuracy of aircraft positioning based on GPS data and without EGNOS (European Geostationary Navigation Overlay Service) correction in ellipsoidal coordinates in the air test in Dęblin.

In Figure 11, the results of the accuracy of aircraft positioning using a GPS system without EGNOS corrections is shown. The results of the accuracy of the GPS system are referenced to the air test in Dęblin. Moreover, the aircraft positions from a GPS single-frequency solution were compared with a dual-frequency PPP method. The accuracy of latitude ranged between +3.1 and +6.4 m. In addition, the mean value of accuracy of latitude equaled +4.8 m, with the RMS error of approximately 4.7 m. The accuracy of longitude ranged between +1.3 and +2.7 m. Furthermore, the mean value of the positioning accuracy of longitude equaled +2.0 m and the RMS error it was 2.1 m. The mean value of the positioning accuracy of the ellipsoidal height equaled −4.2 m, whereas the RMS error equaled 5.3 m. The amplitude of the obtained results of the positioning accuracy along the axis h ranged between −6.9 and −5.2 m. The results in Figure 11 present why the GPS system has limitations in the case of quality of satellite positioning in air transport. If the EGNOS corrections are applied in the observation model of the SPP method, the accuracy of aircraft positioning is improved (see Figure 4). In addition, if only the GPS system is utilized in the observation model of SPP method, then the accuracy of aircraft positioning is reduced. In the case of the air test in Dęblin, EGNOS improved the aircraft position to 78% for latitude, 90% for longitude, and 95% for ellipsoidal height, respectively to the GPS solution. In addition, the RMS parameter from the EGNOS solution ranged from 0.3 to 1.1 m, whereas in the GPS solution it ranged from 2.1 to 5.3 m. Based on the results of the accuracy of aircraft position in Figure 4 and Figure 9, it can be concluded that EGNOS is useful for improving the performance of accuracy parameters in air transport.

**Figure 12:**
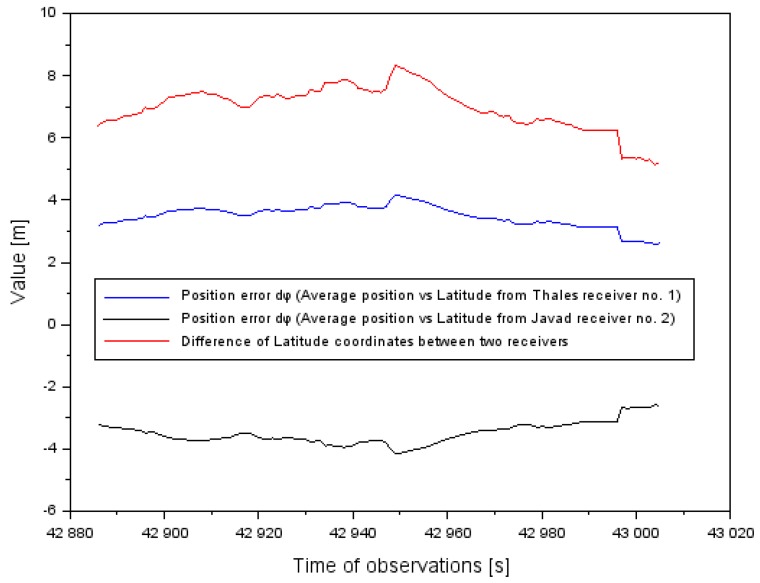
The position error of aircraft coordinates φ in the air test in Chełm.

**Figure 13:**
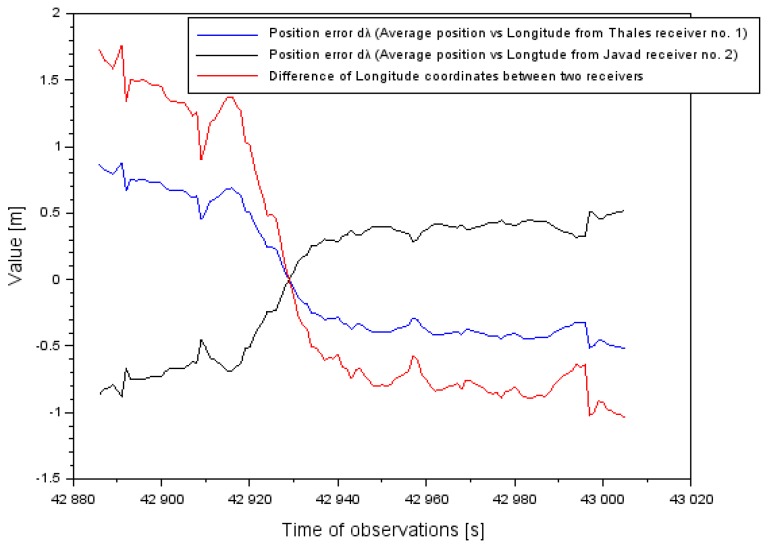
The position error of aircraft coordinates λ in the flight test in Chełm.

**Figure 14:**
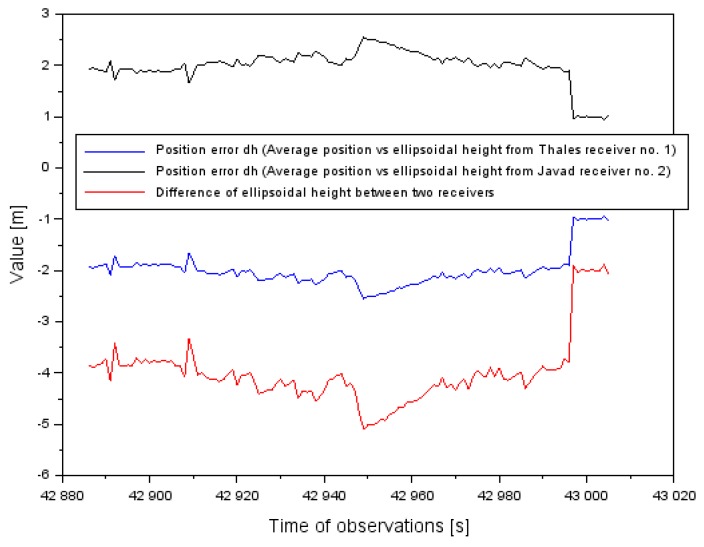
The position error of aircraft coordinates h in the flight test in Chełm.

In general, the additional number of terminations with using EGNOS amendments provides the position of the aircraft with the inspection of conducted navigational calculations. Moreover, such a solution enables the detection and elimination of diverging values of set coordinates. Parameters of the continuity and availabilities can be kept if we have navigation data without breaks from GNSS receiver no. 1, whereas no data can appear in GNSS receiver no. 2. The appointed position of the aircraft from a few GNSS receivers is determined as the resultant position of the movable object. Locating the resultant of the movable object has a key importance for appointing parameter accuracies and credibilities. The advantage of the suggested research methods is an influence on reducing mistakes of position, as presented in Figure 12, Figure 13 and Figure 14. The results in Figure 12, Figure 13 and Figure 14 refer to the air test in Chełm. Figure 12 presents position error dφ (see Equation (3)) in relating the φ coordinate to the difference between two receivers. Based on the results, the absolute value of position error of the φ coordinate was reduced on average about 50%. An arithmetic mean of the absolute value of position error dφ equaled ±3.5 m, whereas the dispersion of the mean difference of the φ coordinate between the two onboard receivers reached 7 m. So, the effect of appointing the position of the resultant is clearly visible.

Figure 13 presents position error dλ (see Equation (3)) in relating the λ coordinate to the difference between two receivers. Based on the results, the absolute value of position error of the λ coordinate was reduced on average by about 50%. The arithmetic mean of the absolute value of position error d λ equaled ±0.1 m, whereas the dispersion of the mean difference of the λ coordinate between the two onboard receivers equaled 0.2 m.

Figure 14 presents position error dh (see Equation (3)) in taking the coordinate h back to the difference between two receivers. Based on the results, the absolute value of position error of the h coordinate was reduced on average by about 50%. The arithmetic mean of the absolute value of position error dh equaled ±2 m whereas the dispersion of the mean difference of the h coordinate between the two onboard receivers equals −4 m.

In [34], the median of standard deviation of the altitude determination was from 1.7 to 4.1 m. In turn, in the presented article in the flight test in Dęblin, the median value of the standard deviation for the determination of the flight altitude was 1.3 m. Therefore, the results in both works are similar and compatible.

In accordance with the recommendations made by the ICAO, the aircraft positioning integrity in the SBAS APV-I and SBAS APV-II procedures in the horizontal plane equaled 16 m. Based on Table 3, the obtained integrity results in the air tests in Dęblin and in Chełm did not exceed the boundary performances for conducting navigation in the LNAV horizontal plane, regarding the ICAO guidelines. In accordance with the recommendations made by the ICAO, the aircraft positioning integrity in the SBAS APV-I and SBAS APV-II procedure, in the vertical plane, equaled 20 and 8 m, respectively. Based on Table 3, the obtained integrity of results in the air tests in Dęblin and in Chełm did not exceed the boundary performance for conducting navigation in the VNAV vertical plane, regarding the ICAO guidelines. In accordance with the recommendations made by the ICAO, the aircraft positioning integrity in the SBAS APV-I and SBAS APV-II procedure, in the vertical plane, was 40 m at most. Based on Table 4, the obtained integrity results in the air tests in Dęblin and in Chełm did not exceed the boundary performance of the HPL parameter in the LNAV vertical plane, for the ICAO guidelines. In accordance with the recommendations made by the ICAO, the aircraft positioning integrity in the SBAS APV-I and SBAS APV-II procedure, in the vertical plane, equaled 50 m and 20 m, respectively. Based on Table 4, the obtained integrity results in the air tests in Dęblin and in Chełm did not exceed the boundary performance of the VPL parameter in the VNAV vertical plane, for the ICAO guidelines. In accordance with the ICAO recommendations, the availability of aircraft positioning in the procedure SBAS APV-I and SBAS APV-II should be higher than 0.99 (or 99%). Based on Table 2 and Table 6, the obtained availability findings in the air tests in Dęblin and in Chełm were higher than the assumed level of 0.99 from the ICAO guidelines. Also, the theoretical number of failures of the EGNOS system during the SBAS APV procedure in the air tests in Dęblin and Chełm equaled C=0.000042÷0.000335 and C=0.000008÷0.000064, respectively. In reality, during the approach to landing procedure at the aerodromes in Dęblin and Chełm, there was no disruption in the operation of the EGNOS system. In view of the above, the technical standards and ICAO recommendations to monitor the positioning of the Cessna 172 in the SBAS APV approach to the landing procedure at the EPDE aerodrome in Dęblin and at the EPCD aerodrome in Chełm were met.

## 5. Conclusions

In the article, the results of numerical research regarding the implementation the EGNOS system within the SBAS APV procedure of landing in aviation are published. The article describes and presents the new solution of the EGNOS system based on the individual position of aircraft from each onboard receiver. The paper describes and discusses the results of an experimental test conducted by the Cessna 172 aircraft at the EPDE military aerodrome in Dęblin. Onboard the Cessna 172 plane, two GNSS receivers (Topcon HiperPro and Thales Mobile Mapper) were installed with the EGNOS positioning function for monitoring changes in the parameters of the aircraft position in real time during the landing phase. The parameters of quality of EGNOS satellite positioning such as accuracy, integrity, continuity, and availability were presented in the paper. Based on the obtained research findings, it was found that the positioning accuracy was not higher than 2.1 m, and the integrity of positioning did not exceed 19 m. Moreover, the availability parameter was found to equal 1 (or 100%); also, no intervals of loss of the continuity of operation of the EGNOS system were recorded. In the paper, the results of the air test from Dęblin were compared with the parameters of positioning quality in the air test conducted in Chełm. The air test in Chełm was also conducted by the Cessna 172 at the Depułtycze Królewskie aerodrome EPCD. On board the Cessna 172, two satellite GNSS receivers with the EGNOS/SBAS positioning function were mounted. Thales Mobile Mapper and Javad Alpha receivers were installed. In the air test in Chełm, the obtained results of parameters of EGNOS quality positioning equaled to better than 4.9 m for accuracy, less than 35.5 m for integrity, 100% for availability, and no breaks in continuity. Based on the results of the air tests in Dęblin and Chełm, it was concluded that the parameters of EGNOS positioning quality in aviation for the SBAS APV procedure were satisfied in accordance with the ICAO requirements.

In this paper, the results of PDOP (Position Dilution of Precision) were also presented and compared from two air tests in Dęblin and Chełm. The maximum results of PDOP amounted to 1.4 in the air test in Dęblin, whereas in Chełm, it equaled 4.0. The geometry of satellites is better in the air test in Dęblin than in the air test in Chełm. The paper also shows how the EGNOS system improved the aircraft position in relation to the GPS-only solution. In this context, the EGNOS system improved the aircraft position about 78% to 95% for each ellipsoidal coordinate axis. In addition, the RMS parameter from the EGNOS solution ranged from 0.3 and 1.1 m, whereas in the GPS solution, it ranged from 2.1 to 5.3 m, respectively.

## Figures and Tables

**Table 1:** Requirements of SBAS APV-I (Satellite Based Augmentation System Approach with Vertical Guidance-I) and SBAS APV-II approach to landing procedure in Polish aviation [3].

Parameter	SBAS APV-I Procedure	SBAS APV-II Procedure
Accuracy	Horizontal accuracy of aircraft position equals 16 m. Vertical accuracy of aircraft position equals 20 m.	Horizontal accuracy of aircraft position equals 16 m. Vertical accuracy of aircraft position equals 8 m.
Integrity	Horizontal integrity of aircraft position equals 40 m. Vertical integrity of aircraft position equals 50 m.	Horizontal integrity of aircraft position equals 40 m. Vertical integrity of aircraft position equals 20 m.
Availability	Availability term is between 0.99 and 0.99999.	Availability term is between 0.99 and 0.99999.
Continuity	Continuity term equals 1 × 10^−6^ ÷ 8 × 10^−6^/15 s.	Continuity term equals 1 × 10^−6^ ÷ 8 × 10^−6^/15 s.

**Table 2:** The values of the availability and continuity terms in the air test in Dęblin.

Parameter	SBAS APV-I Procedure	SBAS APV-II Procedure
Availability	A=1 (or 100%)	A=1 (or 100%)
Continuity	C=0.000042÷0.000335	C=0.000042÷0.000335

**Table 3:** Comparison of accuracy of aircraft position in the air tests in Dęblin and Chełm.

Parameter	Air Test in Dęblin	Air Test in Chełm
Horizontal accuracy for latitude and longitude	−0.7 to +1.7 m	−0.2 to +4.9 m
Vertical accuracy for ellipsoidal height	−1.7 to +2.1 m	−2.8 to –0.7 m
RMS (Root Mean Square) value	0.3 to 1.1 m	0.9 to 4.0 m

**Table 4:** Comparison of integrity of aircraft position in air tests in Dęblin and Chełm.

Parameter	Air Test in Dęblin	Air Test in Chełm
HPL (Horizontal Protection Level) term	2.4 to 13.1 m	22.3 to 35.5 m
VPL (Vertical Protection Level) term	0.1 to 18.8 m	7.1 to 19.2 m

**Table 5:** Comparison of the standard deviation of an average aircraft position in the flight tests in Dęblin and Chełm.

Parameter	Air Test in Dęblin	Air Test in Chełm
$\delta \varphi_{m}$ term	0.1 to 2.0 m	3.6 to 5.9 m
$\delta \lambda_{m}$ term	0.1 to 1.9 m	0.1 to 1.2 m
$\delta h_{m}$ term	0.1 to 3.6 m	1.3 to 3.6 m

**Table 6:** Values of availability and continuity term in the air test in Chełm.

Parameter	SBAS APV-I Procedure	SBAS APV-II Procedure
Availability	A=1 (or 100%)	A=1 (or 100%)
Continuity	C=0.000008÷0.000064	C=0.000008÷0.000064

**Table 7:** The comparison of PDOP (Position Dilution of Precision) values in the air tests in Dęblin and Chełm.

Parameter	Air Test in Dęblin	Air Test in Chełm
Min PDOP	0.9	2.8
Max PDOP	1.4	4.0
Mean PDOP	1.1	3.0

**Table 8:** The relationship between descent angle and accuracy of aircraft positioning in the air tests in Dęblin and Chełm.

Parameter	Air Test in Dęblin	Air Test in Chełm
Minimum values of descent angle (^o^)/Accuracy (m)	0.1°/0.5 m for latitude, −0.7 m for longitude, −1.0 m for ellipsoidal height	0.2°/3.6 m for latitude, −0.2 m for longitude, −2.8 m for ellipsoidal height
Maximum values of descent angle (^o^)/Accuracy (m)	2.7°/1.7 m for latitude, +0.4 m for longitude, +2.1 m for ellipsoidal height	5.6°/4.3 m for latitude, +1.7 m for longitude, −0.7 m for ellipsoidal height
Mean values of descent angle (^o^)/Accuracy (m)	2.2°/1.1 m for latitude, +0.1 m for longitude, +0.3 m for ellipsoidal height	3.9°/3.9 m for latitude, +0.7 m for longitude, −1.7 m for ellipsoidal height
